# Spontaneous Osteonecrosis of the Tarsal Navicular: A Report of Two Cases

**DOI:** 10.1155/2019/5952435

**Published:** 2019-01-30

**Authors:** Yukie Kitaura, Akinobu Nishimura, Shigeto Nakazora, Aki Fukuda, Yoshiyuki Senga, Ko Kato, Akihiro Sudo

**Affiliations:** ^1^Department of Orthopaedic Surgery, Graduate School of Medicine, Mie University, 2-174 Edobashi, Tsu, Mie 514-8507, Japan; ^2^Department of Orthopaedic and Sports Medicine, Graduate School of Medicine, Mie University, 2-174 Edobashi, Tsu, Mie 514-8507, Japan; ^3^Suzuka Kaisei Hospital, 112 Kou, Suzuka, Mie 513-8505, Japan

## Abstract

Spontaneous osteonecrosis of the tarsal navicular, called the Mueller-Weiss syndrome, is an uncommon disease. Patients who are resistant to conservative treatment require operative treatment. However, there is no established operative treatment. Two cases of spontaneous osteonecrosis of the tarsal navicular with double (talonavicular and naviculocuneiform joints) arthrodeses with different locking plates are presented. Removal of necrotic areas from the tarsal navicular and replacement with autologous bone graft procured from the iliac crest followed by arthrodesis using a locking plate were performed. Case 1 was fixed with an LCP Distal Radius Plate (SYNTHES) and 6 2.4 mm locking screws. Case 2 was fixed with a Cervical Spine Locking Plate Variable Angle (SYNTHES) and 4 4.0 mm locking screws. Case 2 achieved solid fusion of the talonavicular-cuneiform joints, but case 1 resulted in nonunion of the talonavicular joint. This difference in internal fixation strength might have caused the difference in the results. Performance of double arthrodeses from the medial aspect using a locking plate is a reasonable operative procedure to treat spontaneous osteonecrosis of the tarsal navicular. Strong primary fixation using a thick plate with large-thread screws was important to obtain joint fusions.

## 1. Introduction

Spontaneous osteonecrosis of the tarsal navicular is quite rare. Nonoperative treatment with an insole is usually sufficient for many patients. However, some cases who fail conservative treatment need operative management. There have been several reports of operative procedures [1–12]. Reported operative techniques include percutaneous drilling decompression, talonavicular arthrodesis, talonavicular-cuneiform arthrodesis, and triple (subtalar and midtarsal joints) arthrodeses according to the severity and/or operator's preference.

In this article, two cases of spontaneous osteonecrosis of the tarsal navicular are described. These two cases had a slightly collapsed tarsal navicular and arthritic changes in the talonavicular and medial naviculocuneiform joints. They were treated by double arthrodeses (talonavicular-cuneiform arthrodesis) with different locking plates.

## 2. Case Presentation

### 2.1. Case 1

A 70-year-old woman presented with a 10-month history of left midfoot pain without any trauma. She was diagnosed as having osteonecrosis of the tarsal navicular based on the findings of plain radiographs from the previous hospital. She was initially treated with an insole. However, the conservative treatment was ineffective for her symptoms. Therefore, surgery was performed.

**Figure 1:**
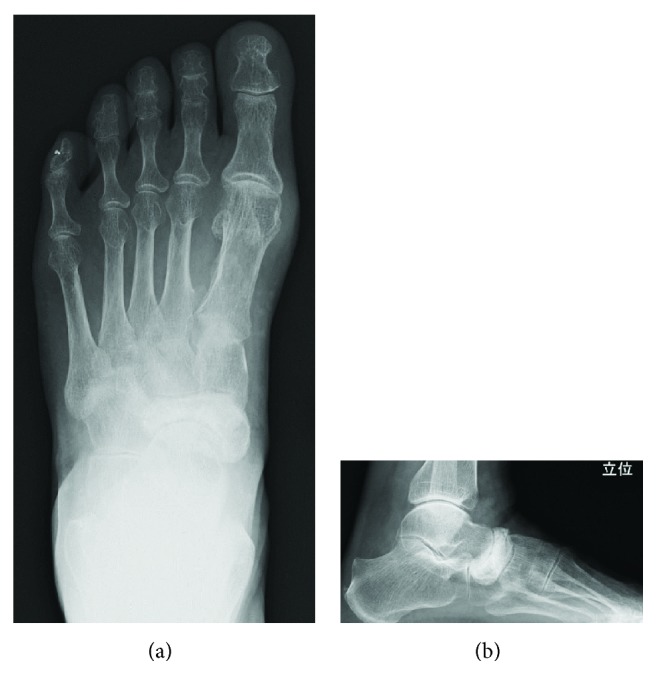
Frontal and lateral radiographs show increased radiodensity, dorsal protrusion, and collapse of the lateral portion of the tarsal navicular.

**Figure 2:**
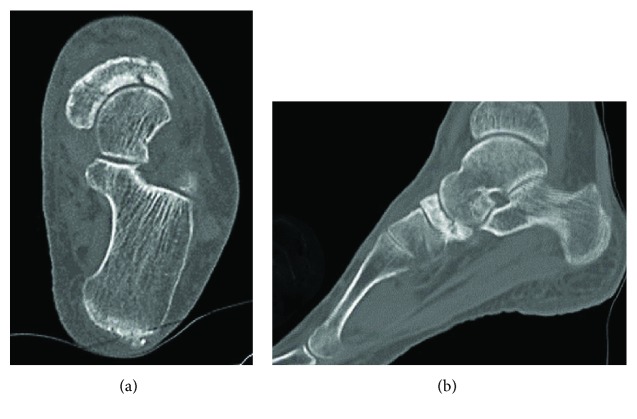
Axial and sagittal CT scans show diffuse sclerosis of the tarsal navicular.

**Figure 3:**
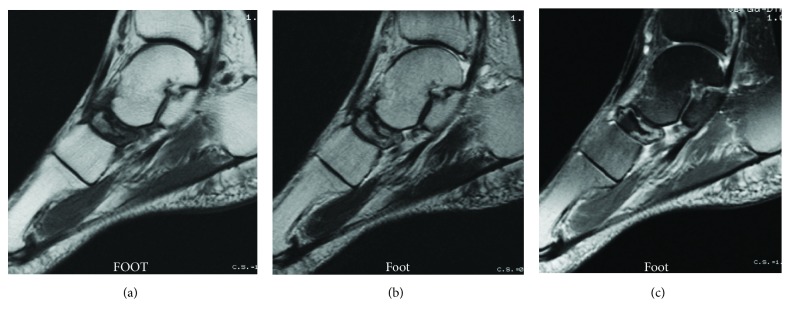
Sagittal MRI shows low signal-intensity areas on both T1-weighted images (a) and T2-weighted images (b) in the marrow of the tarsal navicular. Gd-based MRI (c) shows increased uptake in the peripheral tarsal navicular.

At the time of presentation, her left foot was swollen and had point tenderness at the dorsal side of the talonavicular joint. The preoperative Japanese Society for Surgery of the Foot (JSSF) midfoot scale score [13] was 79 points. Radiographs showed increased radiodensity and dorsal protrusion of the tarsal navicular. Sclerotic collapse was also noted at the lateral aspect of the tarsal navicular (Figure 1). Computed tomography (CT) scans showed diffuse sclerosis and marginal irregularities of the tarsal navicular (Figure 2). Magnetic resonance imaging (MRI) showed low signal-intensity areas on both T1-weighted images and T2-weighted images in the marrow of the tarsal navicular. Gd-based MRI showed increased uptake in the peripheral tarsal navicular, which was representative of hypervascular areas (Figure 3). She was diagnosed with spontaneous osteonecrosis with a Maceira classification of Stage 3 [8].

**Figure 4:**
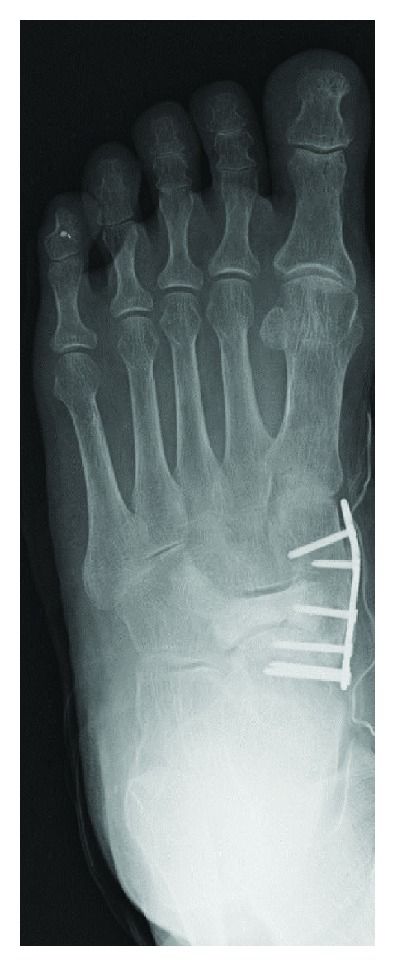
Postoperative radiograph shows fixation using LCP-DRP with 6 2.4 mm locking screws.

**Figure 5:**
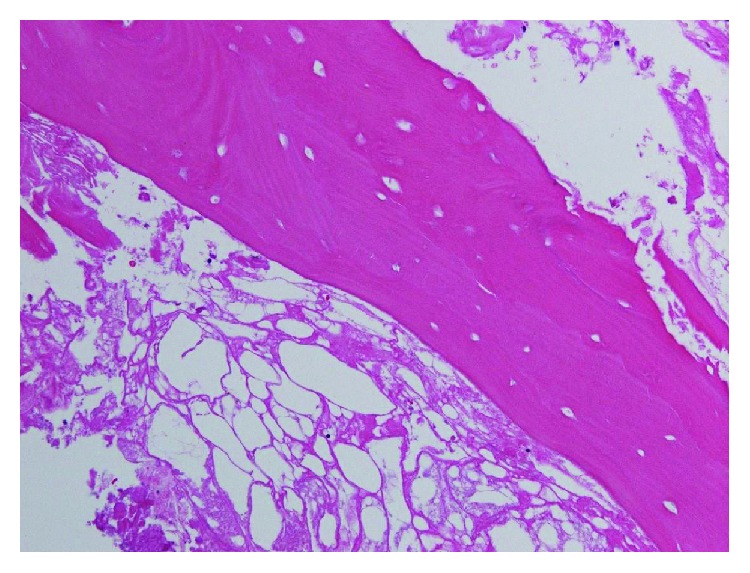
Histopathologic findings show diffuse empty lacunae, suggesting osteonecrosis.

Arthrodeses of the talonavicular and naviculocuneiform joints were selected as the treatment because both joints had cartilage damage on imaging. The articular surfaces of the talus and medial cuneiform that were adjacent to the tarsal navicular and necrotic areas of the tarsal navicular were excised. The blood supply was visible from the marrow of the residual tarsal navicular. The bone defect (5 cm × 1 cm) was reconstructed with a tricortical bone graft harvested from the iliac crest. Arthrodesis was performed using an LCP Distal Radius Plate (SYNTHES) with 6 2.4 mm locking screws from the medial aspect of the foot (Figure 4). Histopathologic findings showed diffuse empty lacunae without any infection, consistent with avascular necrosis (Figure 5). The ankle was immobilized in a cast for 4 weeks after surgery. Partial weight bearing with a patellar tendon-bearing (PTB) orthosis was permitted after the removal of her cast.

**Figure 6:**
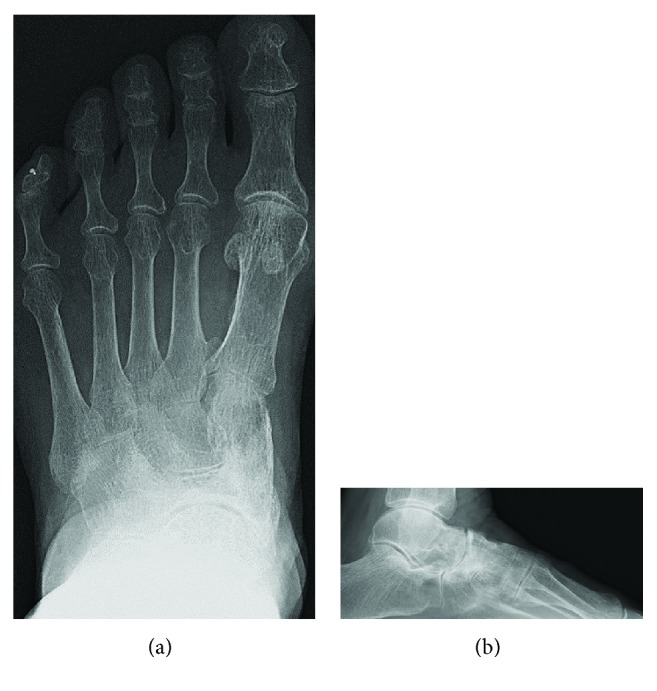
After 5 years of follow-up, radiographs show insufficient fusion of the talonavicular joint.

A radiograph taken 1 year after surgery showed union of the naviculocuneiform joint, but she did not have satisfactory union of the talonavicular joint. The patient had referred pain with the backing out of the most proximal screw. Therefore, the most proximal screw was removed. A radiograph taken 5 years after surgery showed nonunion with mild osteoarthrosis of the talonavicular joint (Figure 6). However, she just had tenderness of the talonavicular joint and had no pain in her usual daily life. The final follow-up JSSF midfoot scale score was 97 points.

### 2.2. Case 2

**Figure 7:**
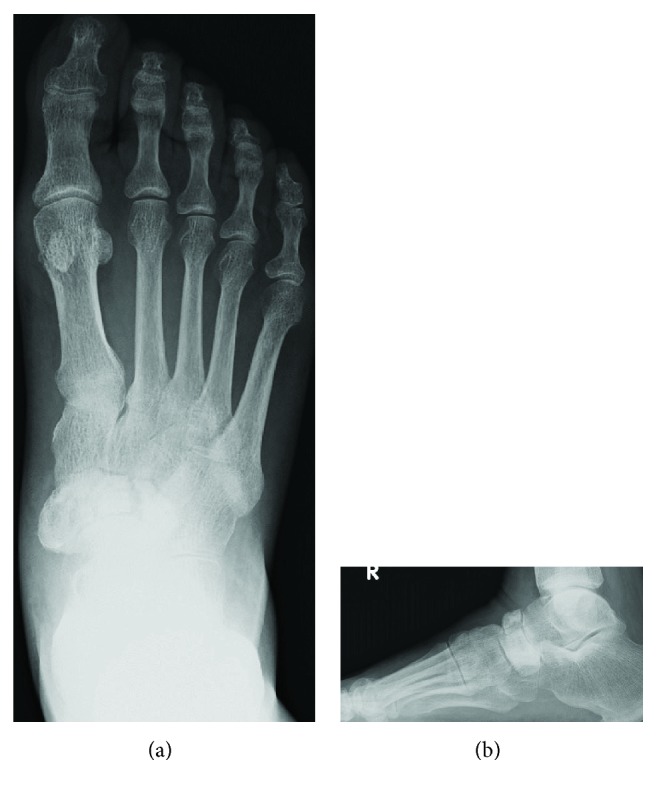
Frontal and lateral radiographs show increased radiodensity, dorsal protrusion, and fragmentation of the tarsal navicular.

**Figure 8:**
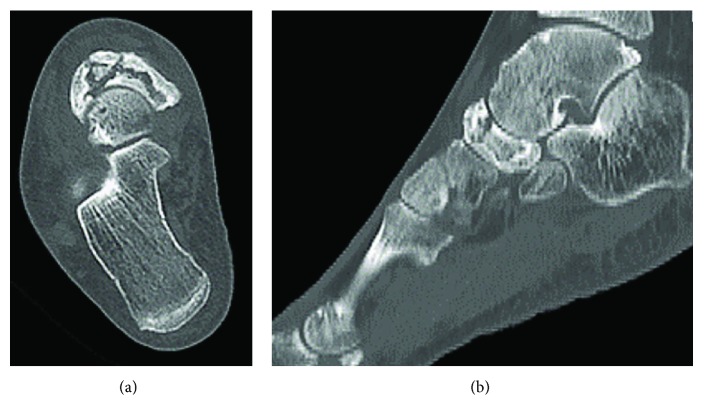
Axial and sagittal CT scans confirm fragmentation of the tarsal navicular and a cystic lesion in the talus.

**Figure 9:**
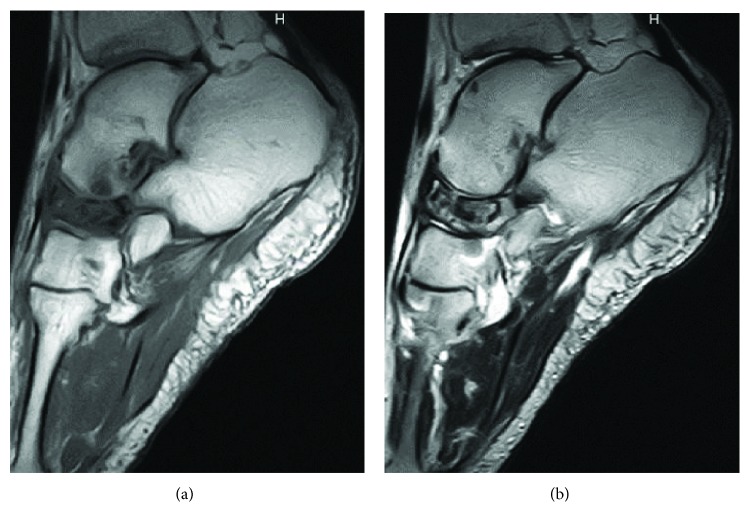
MRI shows low signal-intensity areas on T1-weighted images (a) and T2-weighted images (b) in the marrow of the tarsal navicular.

A 68-year-old woman had right midfoot pain during walking for about 1 year without any trauma. Conservative treatment with an insole was ineffective, so she came to our hospital for surgery. Physical examination showed tenderness and slight swelling at the talonavicular joint. The preoperative JSSF midfoot scale score was 76 points. Radiographs showed increased radiodensity, dorsal protrusion, and fragmentation of the tarsal navicular (Figure 7). CT scans showed a segmented tarsal navicular and cystic lesions with sclerotic changes in the neck of the talus (Figure 8). MRI showed low signal-intensity areas on T1-weighted images and T2-weighted images in the marrow of the tarsal navicular, which suggested osteonecrosis (Figure 9). She was diagnosed with spontaneous osteonecrosis with a Maceira classification of Stage 3 [8].

**Figure 10:**
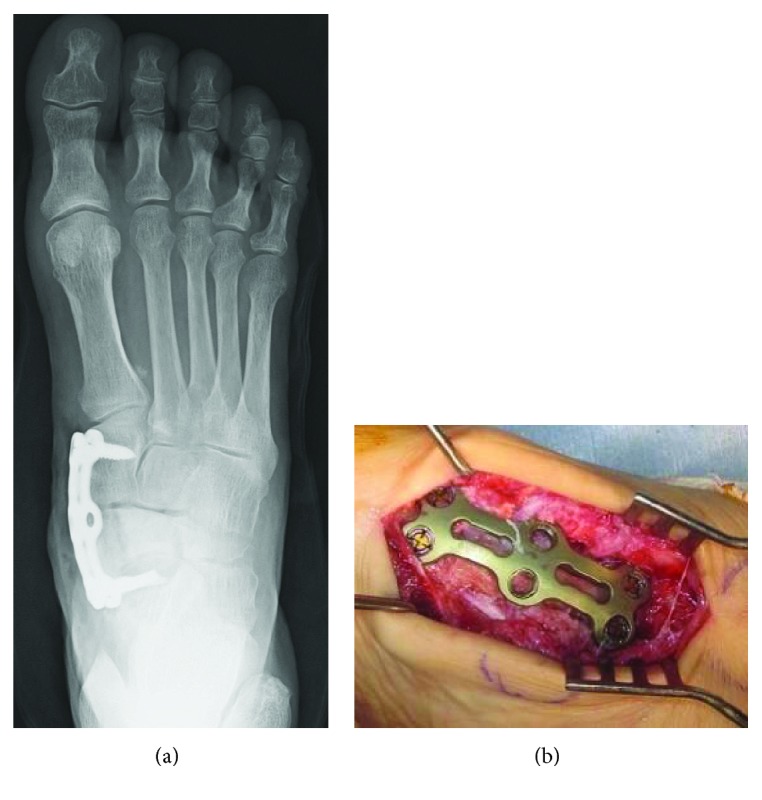
(a) Arthrodeses of the talonavicular and naviculocuneiform joints using CSLP-VA. (b) Postoperative radiograph shows fixation using CSLP-VA with 4 4.0 mm locking screws.

Arthrodeses of the talonavicular and naviculocuneiform joints were planned, as in case 1. A skin incision was made on the medial aspect from the talus to the medial cuneiform. Cartilage delamination of the navicular articular surface was seen. The articular surfaces of the talus and medial cuneiform that were adjacent to the tarsal navicular and the necrotic areas of the tarsal navicular were excised until the blood supply from the marrow of the residual tarsal navicular was visible. The bone defect (4 cm × 1 cm) was reconstructed with a tricortical bone graft harvested from the iliac crest. Primary fixation was performed using CSLP-VA (SYNTHES) with 4 4.0 mm locking screws from the medial aspect of the foot (Figure 10).

**Figure 11:**
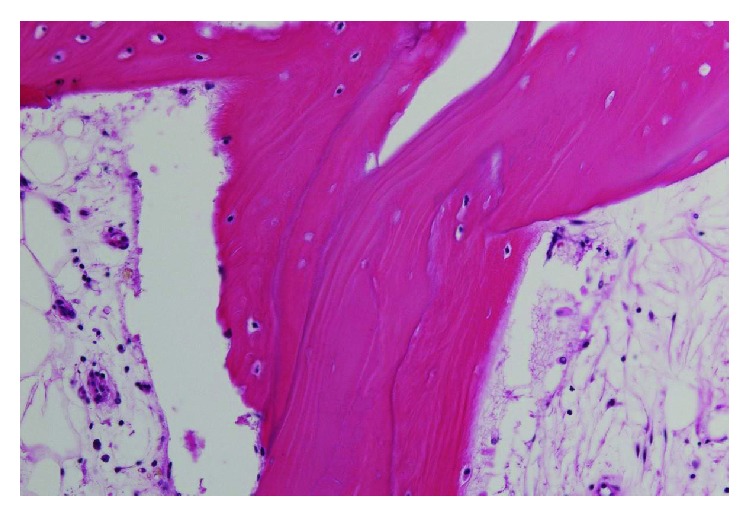
Histopathologic findings show normal osteocytes and empty lacunae.

Histopathological examination showed normal osteocytes and empty lacunae (Figure 11).

**Figure 12:**
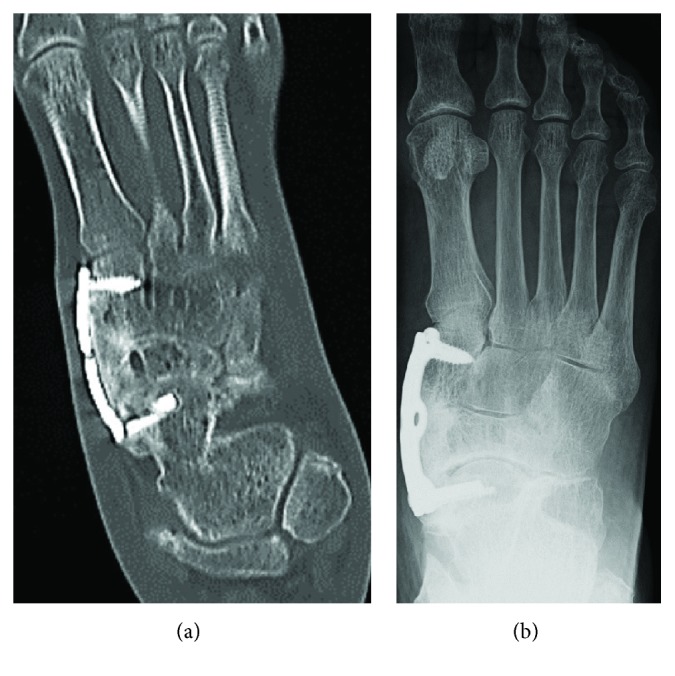
(a) CT scan 6 months after surgery shows sufficient bone union fusion at the talonavicular and naviculocuneiform joints. (b) After 5 years of follow-up, the radiograph shows sufficient fusion.

The same postoperative immobilization and rehabilitation as in case 1 were used. A radiograph taken 6 months after surgery showed sufficient bone union (Figure 12(a)).

Four years after surgery, her radiograph showed complete fusion at the talonavicular and naviculocuneiform joints (Figure 12(b)). The patient was pain-free and could ambulate independently. The final follow-up JSSF midfoot scale score was 100 points.

## 3. Discussion

Spontaneous osteonecrosis of the tarsal navicular, called the Mueller-Weiss syndrome, is an uncommon disease. There have been several cases that underwent operative treatment for spontaneous osteonecrosis of the tarsal navicular. To the best of our knowledge, there have been only 34 cases involving 35 feet (Table 1) [1–12]. In addition, some cases are complicated by the presence of a bipartite tarsal navicular. Therefore, there may be fewer cases.

Spontaneous osteonecrosis of the tarsal navicular is adult-onset avascular necrosis. Secondary osteonecrosis, such as collagen diseases, rheumatoid arthritis, trauma, corticosteroid use, and chronic renal failure, causing osteonecrosis of the tarsal navicular should be ruled out [14]. The precise mechanism of spontaneous osteonecrosis of the tarsal navicular remains unclear. It has been assumed that a chronic disorder of load distribution, with added alteration of foot biomechanics characterized by pes planus, causes disruption of the microvascular system of the tarsal navicular. The tarsal navicular receives its blood supply from 15 to 21 branches of the dorsalis pedis artery and medial plantar artery [15]. They penetrate the circumference of the tarsal navicular radially. The plantar aspect of the bone is supplied by 8 to 9 branches from the medial plantar artery [15]. Therefore, a large part of the tarsal navicular is supplied by the medial plantar artery. Chronic stress to the midfoot, which injures blood flow from branches of the medial plantar artery, might lead to osteonecrosis of the tarsal navicular. The lateral portion of the tarsal navicular is more likely to be affected by a maldistribution of blood flow. It can be seen as a collapse of the lateral portion of the tarsal navicular and increased radiodensity on radiographic findings, and fragmentation of the tarsal navicular and arthritic changes in the midfoot can be seen in progressive cases.

Until the latter half of the 1980s, the patients were treated conservatively [14]. Since the 1990s, operative treatment has been indicated when the patient is resistant to conservative therapy [2]. If left untreated, the disease could lead to advanced midtarsal osteoarthritis and subsequent permanent disability [10]. However, there is no established operative treatment because of the small number of patients and the different severity of each case. Many operative procedures have been described as treatment. The therapeutic strategy for this disease is to restore the length of the medial column of the foot [12]. As operative techniques, percutaneous drilling core decompression [5], talonavicular arthrodesis [1, 2, 11], talonavicular-cuneiform arthrodesis [4, 9, 12], and triple (the subtalar and midtarsal joints) arthrodeses have been reported [7, 11]. The appropriate surgery must be selected for each specific case. Percutaneous core decompression was likely to be selected with no specific findings on radiographs, but characteristic findings such as changes in the intensity of the tarsal navicular can be observed on MRI [5]. For a patient with perinavicular arthritis, talonavicular arthrodesis, talonavicular-cuneiform arthrodesis, and triple arthrodeses are recommended according to severity [4]. Yu et al. described talonavicular-cuneiform joint arthrodesis with an angular stable screw-plate system, with good clinical results [12]. Triple arthrodeses can provide medial and lateral stability [7], but naviculocuneiform arthrosis-related symptoms remain unaddressed [4]. Insufficient fixation may cause nonunion or delayed union. If there are arthritic changes in the subtalar and calcaneocuboid joints on preoperative imaging findings, triple arthrodeses would be required. Treatment using debridement followed by a free medial femoral condyle vascularized bone graft was reported in 2013 [6]. This was reported to achieve satisfactory internal fixation. However, this treatment is highly invasive and requires complicated operative procedures.

The present two cases were classified as stage 3 of the Maceira classification because compression and/or splitting of the tarsal navicular was seen, but Meary-Tomeno's line was neutral. However, these two cases had a slightly collapsed tarsal navicular and arthritic changes in the talonavicular and medial naviculocuneiform joints. Therefore, talonavicular and naviculocuneiform arthrodeses were performed to resolve the osteoarthritic problems in both the talonavicular and naviculocuneiform joints. Cao et al. [3] also reported arthrodeses of the talonavicular and naviculocuneiform joints for the stage 3 Muller-Weiss disease. In the present operations, removal of necrotic areas from the tarsal navicular and replacement with autologous bone graft procured from the iliac crest followed by arthrodeses of the talonavicular and naviculocuneiform joints using a locking plate were performed. Medial aspect fixation was used to support the medial longitudinal arch. In Japan, there was no dedicated plate for fixing in place the area between the talus and the medial cuneiform when these operations were performed. Different plates were used in each case. Case 1 was fixed with an LCP Distal Radius Plate (SYNTHES) and 6 2.4 mm locking screws. Case 2 was fixed with a Cervical Spine Locking Plate Variable Angle (SYNTHES) and 4 4.0 mm locking screws. Case 2 had a successful clinical course with solid fusion, but case 1 had nonunion of the talonavicular joint. The backing out of the most proximal screw appeared to have been gently caused by repeated micromovement due to pseudarthrosis of the talonavicular joint, because we always checked the locking of the screws before finishing the procedures, and the radiograph immediately after the surgery showed no loosening of the most proximal screw. In case 2, a thicker plate and larger thread diameters were used compared to those in case 1; furthermore, the talus of case 2 had 2 screws, while that of case 1 had just 1 screw. Most previous reports did not describe the details of the implants. Three reports used fixation with a plate [9, 11, 12]. Tan et al. reported fixation with a plate of 4.0 mm thickness and 5 3.5 mm/4.0 mm screws, and a good result was achieved [9]. Two reports did not specifically describe the type of plate [11, 12].

In the present cases, case 2 had a good result, and case 1 had a poor result. The difference in internal fixation strength might be the reason for the difference in the results. These results also indicate that strong primary fixation, through the use of a thick plate and large-thread screws, may be mandatory for joint fusion. Further studies are needed to demonstrate the benefit of such an approach.

## 4. Conclusion

The performance of talonavicular and naviculocuneiform arthrodeses from the medial aspect is reasonable treatment for spontaneous osteonecrosis of the tarsal navicular with talonavicular and naviculocuneiform osteoarthritis. Strong primary fixation using a thicker plate and larger thread diameter and screws may be mandatory for joint fusion.

## Figures and Tables

**Table 1:** Details of spontaneous osteonecrosis of tarsal navicular cases and operative methods.

Author	Case number	Average age (y)	Sex	Operation methods	Implant
Aktaş et al. [1]	1 case, 1 foot	50	Female	Talonavicular arthrodesis (single arthrodesis)	2 cannulated cancellous screws
Brian et al. [2]	1 case, 1 foot	25	Female	Talonavicular arthrodesis (single arthrodesis)	Cannulated cancellous screw
Cao et al. [3]	9 cases, 9 feet	48.2 (range 41 to 58)	4 male and 5 female	Talonavicular-cuneiform arthrodesis (double arthrodesis)	3 4.0 mm cannulated screws
Janositz et al. [5]	1 case, 1 foot	18	Female	Percutaneous drilling decompression	No use
Levinson et al. [6]	1 case, 1 foot	25	Male	Internal fixation with a free medial femoral condyle vascularized bone graft	No use
Lui [7]	6 cases, 6 feet	68 (range 47 to 76)	All female	Arthroscopic triple arthrodesis	3 4.0 mm cannulated screws
Tan et al. [9]	1 case, 1 foot	51	Female	Talonavicular-cuneiform arthrodesis (double arthrodesis)	An 8-hole AO low-contact plate with 3.5/4.0 mm screws
Tosun et al. [10]	1 case, 1 foot	43	Male	Internal fixation with an autologous bone graft	Not used
Wang et al. [11]	6 cases, 7 feet	54 (range 45 to 60)	1 male and 5 female	Talonavicular arthrodesis (single arthrodesis) in 5 feet and triple arthrodesis in 2 feet	Talonavicular arthrodesis with 2 compressive screws and triple arthrodesis with plate and screws
Yu et al. [12]	7 cases, 7 feet	55 (range 46 to 62)	1 male and 6 female	Talonavicular-cuneiform arthrodesis (double arthrodesis)	Screws and plate
Present cases	2 cases, 2 feet	69 (range 68 to 70)	2 female	Talonavicular-cuneiform arthrodesis (double arthrodesis)	LCP Distal Radius Plate (SYNTHES) with 6 2.4 mm locking screws and CSLP-VA (SYNTHES) with 4 4.0 mm locking screws
